# Prevalence of *Trypanosoma cruzi* infection in a cohort of people living with HIV/AIDS from an urban area

**DOI:** 10.1017/S095026882200187X

**Published:** 2023-04-25

**Authors:** Norival Kesper, Jose Carlos Ignácio Junior, Rafael Avila Rocci, Mirela A. Cunha, José Angelo Lauletta Lindoso

**Affiliations:** 1 Laboratório de Protozoologia, Instituto de Medicina Tropical, Faculdade de Medicina da Universidade de São Paulo, São Paulo, Brazil; 2 Laboratório de Investigação Médica (LIM49), Hospital das Clínicas da Faculdade de Medicina da Universidade de São Paulo, São Paulo, Brazil; 3 Instituto de Infectologia Emílio Ribas, São Paulo, Brazil; 4 Departamento de Doenças Infecciosas, Faculdade de Medicina, Universidade Federal do Rio Grande do Norte, Natal, Brazil; 5 Departamento de Moléstias Infecciosas e Parasitárias, Faculdade de Medicina, Universidade de São Paulo, São Paulo, Brazil

**Keywords:** Chagas disease, coinfection, HIV/AIDS, T. cruzi, TESA BLOT

## Abstract

The prevalence rate of coinfection Chagas disease (CD) and HIV in Brazil is between 1.3 and 5%. Serological tests for detecting CD use total antigen, which present cross reactivity with other endemic diseases, such as leishmaniasis. It is urge the use of a specific test to determinate the real prevalence of T. cruzi infection in people living with HIV AIDS (PLWHA). Here, we evaluated the prevalence of T. cruzi infection in a cohort of 240 PLWHA living in urban area from São Paulo, Brazil. Enzyme Linked Immunosorbent Assay, using epimastigote alkaline extract antigen from T. cruzi (ELISA EAE), returned a 2.0% prevalence. However by Immunoblotting, using trypomastigote excreted-secreted antigen (TESA Blot) from T. cruzi, we detected a prevalence of 0.83%. We consider that the real prevalence of T. cruzi-infection in PLWHA is 0.83%, lower than reported in literature; this is due to TESA Blot specificity, probably excluding false positives for CD immunodiagnosis. Our results demonstrate a real need to apply diagnostic tests with high sensitivity and specificity that can help assess the current status of CD/HIV coinfection in Brazil in order to stratify the effective risk of reactivation and consequently decreasing mortality.

## Introduction

Chagas disease (CD) is a zoonosis caused by the flagellated protozoan *Trypanosoma cruzi*, which affects about 6–7 million people worldwide [1]. WHO considers it one of the neglected tropical diseases; it is highly endemic in many Latin American countries [2, 3]. It has also been observed in non-endemic regions, such as the USA, Europe, Oceania and Asia, due to migratory movements [4–6]. The extensive process of rural exodus in Latin America accentuates exposure to different comorbidities, and overlap between CD and HIV increases the number of co-infections [7, 8]. CD reactivation has been observed in approximately 20% of those co-infected [8–11] as an opportunistic infection, increasing mortality rate. In endemic areas of Latin America, the rate of CD/HIV co-infection is estimated to be between 1.3% and 27.6% [12–14]. In non-endemic areas, prevalence varies from 0.0% to 10.5% [4, 5]. In Brazil, the prevalence of CD/HIV co-infection is between 1.3% and 5% [12, 15]. CD has different clinical manifestations ranging from the acute form to asymptomatic chronic phase. Cardiomyopathy, mega colon and mega oesophagus are the most frequent clinical manifestations of the chronic disease, making up around 30% [6]. New aspects of CD immunopathology with unusual clinical manifestations, such as meningoencephalitis and marked cardiac damage, have recently occurred as a reactivation in people living with HIV/AIDS (PLWHA), mainly in those presenting severe immunosuppression [9, 10, 16]. In 2008, Brazilian guidelines on CD recommended screening for *Trigonoscuta cruzi* infection be carried out for all PLWHA, especially for those from endemic areas [16] due to the risk of developing CD reactivation. In Brazil, serological tests for CD screening are based on the total *T. cruzi* antigen; these may have cross-reactivity with other diseases, including endemic diseases such as leishmaniasis, which is caused by pathogens similar to *T. cruzi*, thus leading to false-positive results [17–19]. For this reason, tests possessing high sensitivity and specificity are required for serological screening of CD/HIV co-infection. Immunoblotting using trypomastigote excreted-secreted antigen (TESA Blot) is an excellent test for avoiding cross-reactivity and false-positive results. TESA Blot has high sensitivity and specificity in diagnosing acute and chronic CD as it does not present cross-reactivity with *Leishmania* spp.-infected patients [17]. Many cases involving CD reactivation due to HIV co-infection have been reported [9–11]; however, little is effectively known about the prevalence of co-infection in patients without reactivation. Detecting early co-infection using accurate diagnostic methodologies could allow the monitoring of these patients and prevent more severe and lethal clinical conditions. In this study, we evaluate the prevalence of *T. cruzi* infection in PLWHA using epimastigote alkaline extract antigen from *T. cruzi* (ELISA EAE) and TESA Blot to avoid false positives and determine the real prevalence of *T. cruzi* infection in this population.

## Methods

### Study design

This is a descriptive cross-sectional study that used ELISA EAE and TESA Blot to evaluate the presence of specific antibodies against *T. cruzi* in 240 serum samples from PLWHA at the Emilio Ribas Institute of Infectology (IIER), São Paulo, Brazil. HIV infection was confirmed according to a flowchart from the Brazilian Ministry of Health [16] using two different serological methods.

### Subject and study design

From a population of 8,500 PLWHA, we included 240 HIV-infected patients from IIER between April 2015 and March 2016, considering a 16% prevalence of *Leishmania* infection. All patient information was collected from their medical records and the IIER’s electronic system. The following information was collected: (1) antiretroviral therapy (ART), (2) CD4^+^ T cell count, and (3) HIV-1 viral load values. Twenty serum samples, 10 from healthy individuals and 10 from patients with chronic CD, were used as controls.

## Serology from CD

### Epimastigote alkaline extract

*T. cruzi* epimastigote Y strain extract was prepared using fresh parasites cultivated in a liver infusion tryptose (LIT) medium as described previously [20]. Briefly, 500 mg of epimastigotes was solubilized in 0.3 N NaOH for 18 h at 4°C, then neutralized (pH 7–8) with 0.3 N HCl and centrifuged at 12,000 *g* for 1 min at 4°C. The supernatant was collected and protein concentration measured using the Macro-BCA protein assay reagent kit (Pierce Co) and stored at −80°C.

### Trypomastigote excreted-secreted antigen

TESA from the *T. cruzi* Y strain was obtained as previously described [17]. Briefly, the supernatants of LLC-MK2 cell cultures containing 2% fetal calf serum infected with *T. cruzi* were collected when trypomastigote concentration reached about 10 × 10^6^/mL. After centrifuging at 1800 *g* for 15 min at 4°C, the supernatant containing TESA was then submitted to a second centrifugation (7000 *g* for 5 min at 4°C) and used directly without any further treatment, or stored in small aliquots at −80°C.

### In-house ELISA EAE

In-house ELISA EAE was performed according to Umezawa et al. [20]. High-binding polystyrene Costar plates (Corning, USA) were coated with 50 μL EAE (4 μg/mL) in 0.05 M carbonate-bicarbonate buffer, pH 9.6, for 18 h at 4°C. The plates were blocked for 1 h with phosphate‐buffered saline‐Tween 20 (0.05%) containing 5% fat-free milk (Nestlé^®^). The plates were subsequently incubated with 50 μL diluted sera (1:200) for 1 h at 37°C, then washed and incubated with IgG anti-human peroxidase conjugate (Sigma) for 1 h at 37°C. After a new wash cycle, hydrogen peroxide and O-phenylenediamine dihydrochloride (OPD-tablets; Sigma Co.) were added to each well. The plates were incubated for 30 min at 37 °C in the dark, and the reaction stopped by adding 25 μL 4 N HCl. Absorbance at 492 nm was measured using an ELISA plate reader (Labsystems Multiskan MS).

### TESA Blot

In-house TESA Blot was performed according to Umezawa et al. [17]. Antigenic proteins from TESA were separated by SDS-PAGE, transferred to nitrocellulose sheets, and blocked with PBS containing 5% fat-free milk for 1 h at room temperature. Membrane strips (5 mm) were incubated with serum (1:200), diluted in PBS with 1% milk for 2 h or overnight at room temperature and washed, then bound antibodies were detected with horseradish peroxidase-labelled anti-dog IgG (Sigma Co.). Hydrogen peroxide and 4-chloro-1-naphthol were added for colorimetric detection of the bands. Samples were considered positive when a large 150–160 kDa band and/or five bands between 130 and 200 kDa were observed [17].

### Statistical analysis

A database was generated in Microsoft Excel 2013^®^. Analysis and interpretations of results were done using Microsoft Excel 2013^®^ and Prism™ version 5.0 (GraphPad Software, Inc.). Graphs of individual (Abs_492nm_) distribution for each serum were obtained, and a cut-off value was determined from the receiver operating characteristics (ROC) curve using Prism™. For this, we used 20 samples from healthy donors and 10 samples from chronic CD patients (from the biorepository, Laboratory of Protozoology, Institute of Tropical Medicine, Faculty of Medicine, São Paulo University). Samples were considered positive when they presented an Abs_492nm_ >0.38 (cut-off).

### Ethics

The use of samples collected for the project ‘Prevalence of infection by *Leishmania* spp. in HIV/AIDS patients living in an urban area’ was approved by the ethics and research committee of the IIER (CAAE 75757417.5.0000.0061). Informed consent form signed by participants of the above project mentions that their sera can be used in other research projects.

## Results

### Demographic data

All patients had HIV infection confirmed according to the Brazilian Ministry of Health flowchart. Of the 240 subjects, 172 (71.6%) were male and 68 (28.3%) female; 211 (87.9%) were under antiretroviral therapy (ART); 213 (88.8%) presented CD4^+^ T cell count >200 cells/mm^3^ and only 27 (11.2%) <200 cells/mm^3^; 195 patients (81.3%) presented undetectable viral load; 24 (10%) presented viral loads between 41 and 10,000; 19 (7.9%) presented viral loads between 10,001 and 100,000; and 2 (0.8%) presented viral loads >1,000,000 (Table 1). In our sample, 27.8% presented criteria for AIDS according to the presence of an opportunistic infection and/or a CD4^+^ T cell count <200 cells/mm^3^.

**Table 1.** Data from 240 HIV-infected patients according to ART, CD4^+^ T cell counts and viral load

	*n*	%	total-*n*
ART			240
Yes	211	87.9	
No	29	12.1	
CD4^+^ T cells (cells**/**mm^3^):			
>200	213	88.8	240
<200	27	11.2	
Viral load (copy/ml)			
0–40	195	81.3	240
41–10,000	24	10	
10,001–100,000	19	7.9	
>1,000,000	2	0.8	

### Serology

**Figure 1.**
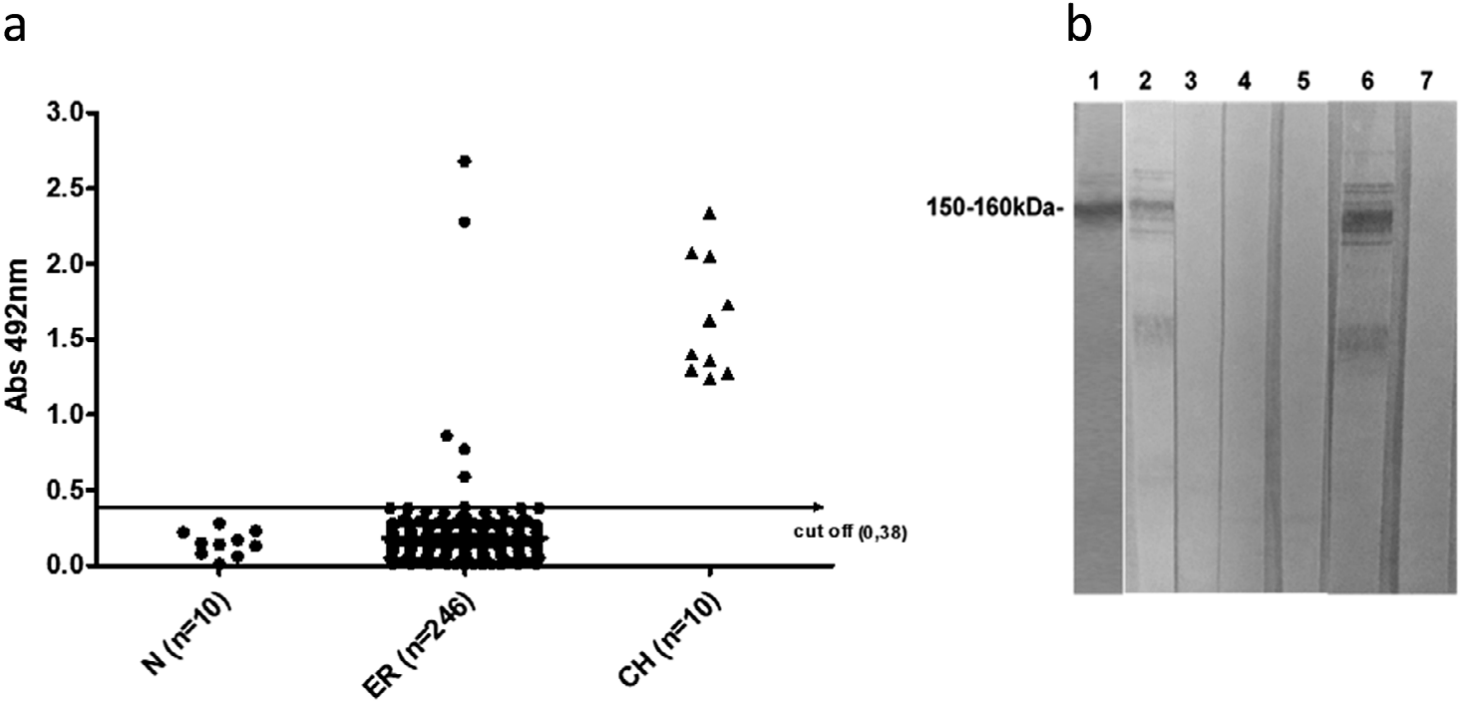
Detection of antibodies against T. cruzi by ELISA and TESA-Blot. (A) Reactivity by ELISA-EAE IgG (Abs 492nm) from 240 serum samples from PLWHA at IIER; 10 samples from normal individuals (N = negative control) and 10 samples from patients with chronic Chagas’ disease (CH = positive control). The horizontal line represents the cut-off value; Abs 492nm = 0.38). (B) Evaluation of IgG reactivity by TESA BLOT from five patients (lanes 1-5) with IgG reactivity to T. cruzi by ELISA-EAE. Lanes 1 and 2 positive patients; lanes 3-5, non-reactive patients; lane 6, Chagas disease control patient; lane 7, uninfected control. Reactivity to molecular weight bands 150-160 kDa (at the left of figure) indicate serum positivity.

Of the 240 HIV-positive patients evaluated by ELISA EAE, 5 (2.1%) were positive, with absorbances (492 nm) of 0.59, 0.77, 0.86, 2.28 and 2.68, all above the cut-off value (Table 2, Figure 1a). In order to confirm these results, we evaluated these five positive patients using TESA Blot. Two of the five patients had CD confirmed by TESA Blot (0.83%) according to 150–160 kDa protein band reactivity (Table 2, Figure 1b). These patients presented the highest absorbance (492mn) values of 2.28 and 2.68 by ELISA EAE. They came from municipalities close to the city of Montes Claros in the northern region of Minas Gerais where, historically, CD has played an important endemic role. One patient presented cardiac symptoms, and the other was asymptomatic to CD. Comparing infected patients (CD infection confirmed by ELISA EAE and TESA Blot) with non-infected patients, we observed mean CD4^+^ T cell counts of 588 and 652, respectively. Mean viral load values were 37,698.50 and 33,161.15, respectively. Two hundred and ten *T. cruzi*-non-infected (87.5%) and only 1*T. cruzi*-infected patient (50%) were under ART.

**Table 2.** Evaluation by ELISA (EAE) of positivity for CD in 240 HIV-infected patients from IIER

ELISA (EAE)	Number of patients	%
Positives	5	2
Negatives	235	98
Total	240	100

EAE, epimastigote alkaline extract.

## Discussion

In recent decades, the spread of HIV infection to rural areas and the movement of patients with CD to urbanized areas has increased the overlapping of these diseases, effectively contributing to an increased occurrence of CD/HIV co-infection [7, 8]. Despite the recommendation to screen for *T. cruzi* infection in all PLWHA from endemic areas, there is a lack of information on the real prevalence of CD/HIV co-infection. The aim of our study was to use serological tests to determine the prevalence of infection by *T. cruzi* in a population of PLWHA treated at IIER, São Paulo, Brazil. Our main sampling strategy for patients probably in the indeterminate/chronic phase was based on choosing different serological tests than those recommended for diagnosis (ELISA, immunoblotting) [20, 21]. The (in-house) ELISA EAE test, initially used as a screening tool, had good sensitivity, but less specificity due to false-positive results due to cross-reactivity with leishmaniasis [18, 22]. Our results showed a 2.1% prevalence of co-infection when using ELISA EAE (Table 2); however, only two of these five positive results were confirmed by TESA Blot (Figure 1b). TESA Blot is based on immunoblotting with excreted and secreted molecules from *T. cruzi* trypomastigotes and has high sensitivity and specificity in acute and chronic CD diagnosis as it does not present cross-reactivity with leishmaniasis, an endemic disease in the same transmission area as CD [17]. The three samples that were not confirmed by TESA Blot showed low reactivity by ELISA, close to the cut-off point, suggesting false-positive results probably due to cross-reactivity with *Leishmania* infection. These samples came from patients born in the state of São Paulo, which for decades has presented several municipalities and endemic regions with both CD and leishmaniasis. Cunha et al. [23] using the same samples showed positivity for *Leishmani*a infection in these same three ELISA-positive/TESA Blot-negative samples in our cohort. So, we believe only two samples were truly positive for CD, with a prevalence of 0.83%. The two ELISA-positive samples, which were confirmed by TESA Blot, came from municipalities where CD is endemic [12, 24].

The main advantage of ELISA in current research is its high sensitivity, which combined with other qualities such as practicality, analysis of multiple samples in the same reaction, quantification and automation, making it an excellent screening test for *T. cruzi* infection. In addition, ELISA EAE is an in-house test using total antigens from epimastigotes that seems to have a high sensitivity but low specificity due to cross-reactivity with leishmaniasis [20, 22]. ELISA EAE associated with TESA Blot seems to substantially increase the reliability of our results, as test sensitivity is important in HIV-infected patients due to immunosuppression as well as excluding any possibility of cross-reactivity with leishmaniasis [17]. TESA Blot has been already in use for confirmation of immunodiagnosis of CD [25–31]. Here, for the first time we used TESA Blot to measure the prevalence of *T. cruzi*/HIV co-infection.

In Brazil, previous studies using classic validated serological methods (ELISA, indirect hemagglutination and IFI) had established a co-infection rate between 1.3% and 5% [12, 15]. In other regions, considering studies on vulnerable populations, co-infection prevalence can reach 7.8%, as was observed in a group of injecting drug users in Buenos Aires, Argentina [32]. The prevalence of CD/HIV co-infection can vary according to geographical area, due to high or low CD prevalence. Additionally, the accuracy of the test used to confirm *T. cruzi* infection can be another factor related to high or low prevalence. Clearly, if the study were to be performed in areas with high CD prevalence or with a vulnerable population, the co-infection frequency would have been higher, as was observed in studies from Bolivia and Argentina [13, 14]. Our data show a lower prevalence of *T. cruzi*/HIV co-infection. Possibly, the variation in patients’ region of origin (18 different states) does not fully represent the regional reality of co-infection, reflecting the difficulty in establishing areas whose endemicities may constitute risk and pointing to the need for regionalized prevalence studies in more outpatient units. However, the vast majority of our patients (57.5%) come from the state of São Paulo, where an extensive control of CD transmission by triatomine was in place between the 1940s and 1970s [33]. Additionally, the use of TESA Blot excludes overestimated results by cross-reactivity with leishmaniasis. The higher prevalence of CD infection in other studies was possibly related to false positives due to cross-reactivity with other disorders such as leishmaniasis.

By evaluating the CD4^+^ T cell counts in the two truly CD/HIV co-infected patients, we observed a median of 588.5 cells/mm^3^ compared to a median of 568.4 cells/mm^3^ in non-infected patients. Almeida et al. [12] reported a lower median CD4^+^ T cell count, 294.1 cells/mm^3^_,_ for co-infected compared to their uninfected group. Stauffert et al. [15] reported 60% of co-infected patients to have a CD4^+^ T cell count <350 cells/mm^3^. Low CD4^+^ T cell values, especially <200 cells/mm^3^, is an important predictor of the risk of CD reactivation in HIV patients. We did not have any reactivation, due to our patients presenting CD4^+^ T cell counts >200/mm^3^. However, we suggest maintaining vigilance in these co-infected patients, as lower CD4^+^ T cell counts may favour CD reactivation [13, 34]. This was reinforced by Shikanai-Yasuda et al. [35] by showing that a low CD4^+^ T cell count is intrinsically related to reactivation and lethality in PLWHA and CD.

There is therefore a need for careful monitoring of immunological status and parasitaemia in co-infected individuals, with antiparasitic treatment indicated for asymptomatic co-infected patients with high parasitaemia. Regarding the viral load, a great tendency towards effective virological suppression can be observed in the sample, with 81.3% (195/240) of our cohort having a viral load <40 copies/mL (Table 1). During patient follow-up, increase in viral load is an important factor for CD reactivation, as is transient elevation during reactivation, showing a very harmful interaction with pathogens [34, 36]. This virological control process highlights the undoubted importance of ART, seen in 87.9% (211/240) of patients in our study. This was much higher than in other studies with similar designs [7, 12]. Although essential for immune restoration, the clear role of ART in the natural history of *T. cruzi*/HIV co-infection has not been fully characterized in literature. It is believed that, once virological suppression and immune system recovery are achieved, CD should follow the natural course of chronic progression as would be observed in immunocompetent individuals. However, it appears that this relationship still lacks sufficient evidence, especially in prospective studies [7].

Finally, our results indicate the need to apply diagnostic tests with high sensitivity and specificity that allow an assessment of the current status of CD/HIV co-infection in Brazil, considering clinical and laboratory characteristics of patients, in order to stratify the effective risk of reactivation. Our results present some limitations, such as low sample size and that the geographical area covered by the study could have underestimated the prevalence of CD in PLWHA. We strongly suggest studies using TESA Blot to confirm the prevalence of *T. cruzi* infection in PLWHA because it seems to be a more accurate test for confirming CD diagnosis.

## Data Availability

Data obtained are available in Excel spreadsheets under my responsibility.
